# Conditioned Medium from Irradiated Keratinocytes as a Tool for Investigating Communication within the Melano‐epidermal Unit

**DOI:** 10.1002/cpz1.70428

**Published:** 2026-07-27

**Authors:** Lais Zortéa, Larissa Gomes Gusmão de Almeida, Daza de Moraes Vaz Batista Filgueira, Ana Paula de Souza Votto

**Affiliations:** ^1^ Laboratório de Cultura Celular, Instituto de Ciências Biológicas Universidade Federal do Rio Grande – FURG RS Brasil; ^2^ Programa de Pós‐Graduação em Ciências Fisiológicas, Instituto de Ciências Biológicas Universidade Federal do Rio Grande – FURG RS Brasil

**Keywords:** keratinocyte‐conditioned medium, melano‐epidermal communication, melanocyte response, skin photobiology, ultraviolet radiation, UVA, UVB

## Abstract

Keratinocytes and melanocytes form the melano‐epidermal unit, whose communication is essential for maintaining the integrity, homeostasis, and functionality of the skin. This interaction is mediated by keratinocytes’ release of various soluble factors, which modulate key processes in melanocytes such as melanogenesis, proliferation, adhesion, and cellular differentiation. Exposure of keratinocytes to ultraviolet (UV) radiation can induce changes in their secretion profile, directly influencing the response of melanocytes and, consequently, skin pigmentation and protection against environmental aggressions. In this article, the presented protocol was developed and validated for obtaining conditioned medium from keratinocytes irradiated with UVA or UVB, followed by evaluation of cell viability as a criterion to determine the maximum applicable radiation dose. The conditioned medium obtained was applied to melanocyte cultures to investigate the effects mediated by secreted factors following radiation exposure. The methodology presented constitutes a tool for studying cellular communication under environmental stress conditions and may be used for developing photoprotective agents, for deepening understanding of keratinocyte‐melanocyte interactions following UV radiation, and for contributing to the elucidation of mechanisms regulating pigmentary disorders. © 2026 The Author(s). *Current Protocols* published by Wiley Periodicals LLC.

**Basic Protocol**: Preparation, UV irradiation, and handling of conditioned medium from keratinocytes and application to melanocyte cultures

## INTRODUCTION

Keratinocytes, the most common cells of the epidermis, play a fundamental role in maintaining the integrity and functionality of the skin (Junqueira & Carneiro, 2017). In addition to forming the skin barrier, they are responsible for regulating the behavior of neighboring cells, such as melanocytes, through the release of signaling molecules (Upadhyay et al., 2021). Melanocytes are the skin cells responsible for melanin production (melanogenesis), and with keratinocytes, they form the melano‐epidermal unit (Fitzpatrick & Breathnach, 1963).

The melano‐epidermal unit is responsible for skin pigmentation, whereby a melanocyte produces melanin and delivers it to adjacent keratinocytes (Abdel‐Malek & Kadekaro, 2006). Keratinocytes are responsible for modulating melanogenesis in melanocytes, being able to induce either an increase or a decrease in melanin synthesis, as well as alter mechanisms involved in the secretion and delivery of pigment granules in the skin. In addition to melanogenesis, keratinocytes also modulate the proliferation, adhesion, and differentiation of melanocytes through the secretion of cytokines, growth factors, and other soluble mediators, making intercellular communication between the cell types essential for epidermal homeostasis (Dainese‐Marque et al., 2024; Li et al., 2024).

The interaction between these two cell types can be altered by environmental stimuli, the main one being exposure to ultraviolet (UV) A (UVA) and B (UVB) radiation. According to the literature, keratinocytes tend to induce an increase in melanogenesis in response to radiation exposure (Gilchrest et al., 1996). However, despite the constant contact of these cells with this stressor, little is known about how other responses induced by keratinocytes are altered in melanocytes following UVA and/or UVB irradiation.

Understanding keratinocyte‐melanocyte communication following UV exposure is essential for comprehending the physiology of the skin and its responses to stress. To better understand the mechanisms involved in these responses, cell culture models containing keratinocytes and melanocytes are crucial. However, the currently available *in vitro* methods generate complex responses, requiring an experimental approach capable of considering the various factors released during this communication.

Thus, the use of conditioned medium from irradiated keratinocytes represents a relevant approach to investigate how the factors released by these cells impact the physiology of melanocytes after exposure to the stressor. Standardizing the parameters for the production of this medium, such as radiation dose and post‐irradiation incubation time, is essential to ensure data reproducibility and the reliability of the *in vitro* models used.

Although conditioned medium approaches and UV irradiation models have been previously employed to investigate skin cell responses, detailed methodological descriptions are often lacking, which may hinder reproducibility across laboratories. Therefore, the aim of our work was to develop and validate a standardized protocol for producing conditioned medium from UVA‐ and UVB‐irradiated keratinocytes, followed by cellular proliferation assessment. After defining the highest radiation dose that did not compromise keratinocyte viability, the conditioned medium obtained was applied to melanocyte cultures. By providing a detailed, step‐by‐step methodology, including cell culture conditions, irradiation procedures, conditioned medium collection, and application to melanocytes, the protocol presented here offers a reproducible and adaptable tool for investigating keratinocyte‐melanocyte communication. Furthermore, it may serve as a basis for future studies evaluating epidermal responses to environmental stimuli and testing compounds with photoprotective or signaling‐modulating potential between keratinocytes and melanocytes.

## STRATEGIC PLANNING

**Figure 1:**
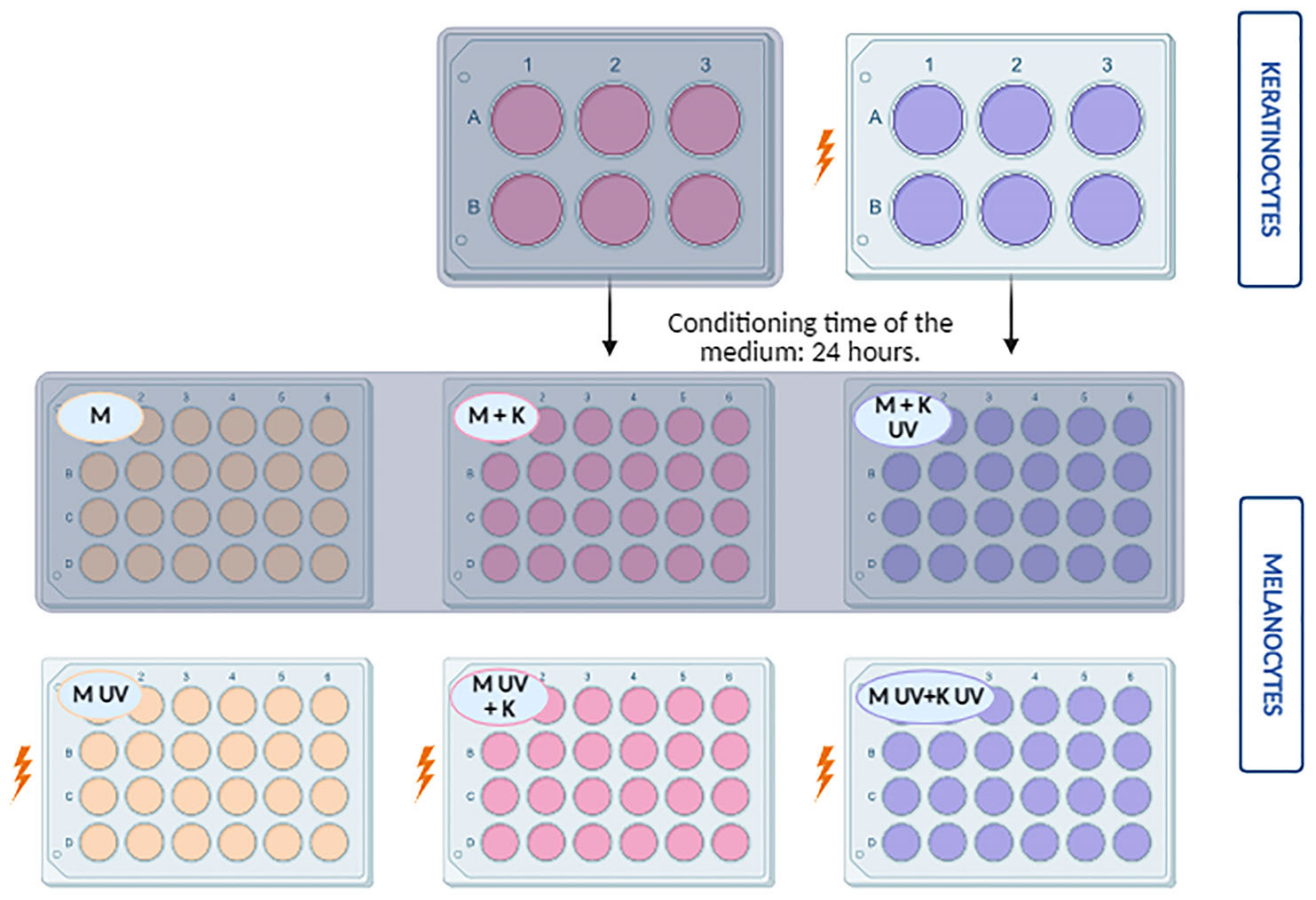
Illustrative diagram of the culture conditions used in the experimental protocol. Keratinocytes were cultured in 6‐well plates and irradiated or not irradiated with UV. After 24 hr, conditioned media were collected. Melanocytes were cultured in 24‐well plates under different conditions according to the experimental group: melanocytes only (M), melanocytes with conditioned medium from non‐irradiated keratinocytes (M‐KCM), melanocytes with conditioned medium from irradiated keratinocytes (M‐iKCM), UV‐irradiated melanocytes (iM), UV‐irradiated melanocytes with conditioned medium from non‐irradiated keratinocytes (iM‐KCM), and UV‐irradiated melanocytes with conditioned medium from irradiated keratinocytes (iM‐iKCM). The lightning symbol indicates an irradiated condition.

*NOTE*: All steps of the protocol must be performed in a sterile environment, using a laminar flow hood (Labconco, cat. no. 021125411H).

The following protocol considers the production of conditioned medium for use in an experiment with six 24‐well plates of melanocytes, with one for each of the following groups: melanocytes in culture medium (M), UV‐irradiated melanocytes in culture medium (iM), melanocytes in non‐irradiated keratinocyte‐conditioned medium (M‐KCM), UV‐irradiated melanocytes in non‐irradiated keratinocyte‐conditioned medium (iM‐KCM), melanocytes in UV‐irradiated keratinocyte‐conditioned medium (M‐iKCM), and UV‐irradiated melanocytes in UV‐irradiated keratinocyte‐conditioned medium (iM‐iKCM) (Fig. 1). The plate setup is provided in this way for educational purposes, but the volume of medium and the selection of groups can be adjusted according to the needs of the analysis to be performed, as long as the keratinocyte concentration in the plates is maintained.

## PREPARATION, UV IRRADIATION, AND HANDLING OF CONDITIONED MEDIUM FROM KERATINOCYTES AND APPLICATION TO MELANOCYTE CULTURES

This protocol describes a 3‐day procedure for plating keratinocytes and melanocytes, exposing both cell types to controlled doses of UV radiation (UVA, UVB), generating keratinocyte‐conditioned medium, and applying it to melanocyte cultures. Keratinocytes are first seeded and irradiated under defined conditions, followed by a 24‐hr incubation to allow secretion of soluble factors into the culture medium. The conditioned medium is then collected, filtered, and used to treat melanocytes, which may also undergo UV irradiation depending on the experimental group.

If performed correctly, the protocol results in adherent, viable monolayers of both cell types; reproducible UV exposure based on calculated irradiance and dose; and sterile, cell‐free conditioned medium suitable for downstream analyses. Consistency in cell density, irradiation parameters, and sterile handling is essential to ensure experimental reliability and reproducibility.

### Materials

RPMI‐1640 culture medium (Gibco, cat. no. 23400‐021)
Fetal bovine serum (FBS; Gibco, cat. no. 12657‐029)
Antibiotic‐antimycotic solution (Gibco, cat. no. 15240‐062)
HaCaT keratinocytes
Phosphate‐buffered saline (PBS; pH 7.4; see recipe)
RPMI‐1640 culture medium with 30% (v/v) FBS and 2% (v/v) antibiotic‐antimycotic solution
Melan‐a melanocyte cell line

Beakers, sterile
6‐well plates (Kasvi, cat. no. K12‐006)
37°C, 5% CO_2_ incubator (Ultrasafe, cat. no. 1011R0485CE)
Pipets, sterile
Photoprotective material (e.g., culture plate lid covered with opaque black tape)
Irradiation chamber
UVA lamp (Vilber, cat. no. VL‐115L)
UVB lamp (Vilber, cat. no. VL‐115M)
24‐well plates (Kasvi, cat. no. K12‐024)
Sterile containers (e.g., sterile beakers or conical centrifuge tubes)
0.22‐µm PES syringe filters (Kasvi, cat. no. K18‐PES‐ESTÉRIL)
Syringes, sterile (e.g., 5‐ml syringes, Descarpack, cat. no. 10330669025)

Additional reagents and equipment for selected analysis of melanocytes at desired time points.

#### Setting up the keratinocyte plates

1 In a sterile beaker, prepare the culture medium: RPMI‐1640 culture medium supplemented with 15% (v/v) FBS and 1% (v/v) antibiotic‐antimycotic solution.
  For a total of 110 ml solution, combine 92.4 ml RPMI‐1640, 16.5 ml FBS, and 1.1 ml antibiotic‐antimycotic solution.
2 Set up 6‐well plates with HaCaT keratinocytes at a concentration of 2 × 10^5^ cells/ml in 3 ml medium per well.
  This seeding concentration ensures the cells are at ∼60% confluence after attachment and ∼90% confluence at the time of conditioned medium collection.
  The calculation of the initial cell culture volume to use is done using the following formula: C1.V1 = C2.V2, where C1 = initial concentration; V1 = initial volume; C2 = final concentration; V2 = final volume.
3 Incubate the plates in a 37°C, 5% CO_2_ incubator for 24 hr to allow adherence.
  Ensure that the plates are level in the incubator to avoid uneven distribution of cells in each well.

#### Irradiation of the keratinocytes

4 After 24 hr of incubation, transfer the 6‐well plates to the laminar flow hood, maintaining sterile conditions.
5 Using separate sterile pipets, remove the culture medium from each well individually and wash the wells two times each with 2 ml PBS (pH 7.4).
  The washing should be done well by well, adding PBS slowly to the wall of the well to avoid detaching the cells. Carefully remove the PBS after each wash, touching the tip of the pipet to the well intersection to avoid removing the cells.
6 Add 1.5 ml RPMI‐1640 culture medium without FBS and without antibiotic‐antimycotic solution (pure medium) to each well.
7 Cover plates that will be used as controls, i.e., conditioned medium from non‐irradiated keratinocytes, with photoprotective material, such as a culture plate lid covered with opaque black tape, before placement in the irradiation chamber (see step 8).
8 Place the plates in the irradiation chamber with UVA and UVB lamps. Program the equipment according to the desired UV radiation dose to be used (UVA or UVB).
  Before irradiation, calculate the exposure time using the following formula: time (minutes) = 1000 × dose (J/cm²) / 60 × irradiance (mW/cm²) (Diffey, 2002).
9 After the desired radiation dose is reached, add 1.5 ml RPMI‐1640 culture medium with 30% FBS and 2% antibiotic‐antimycotic solution to each well, without removing the previous medium.
  This ensures a final volume of 3 ml, with final concentrations of 15% FBS and 1% antibiotic‐antimycotic solution.
10 Incubate the plates again in a 37°C, 5% CO_2_ incubator for 24 hr.

#### Setting up the melanocyte plates

11 In a sterile beaker, prepare fresh culture medium as in step 1.
  For a total of 150 ml solution, combine 126 ml RPMI‐1640, 22.5 ml FBS, and 1.5 ml antibiotic‐antimycotic.
12 Set up 24‐well plates with the melan‐a melanocyte cell line at a concentration of 5 × 10^4^ cells/ml in 1 ml medium per well.
  The calculation of the initial cell culture volume to use is done using the following formula: C1.V1 = C2.V2, where C1 = initial concentration; V1 = initial volume; C2 = final concentration; V2 = final volume.
13 Incubate the plates in a 37°C, 5% CO_2_ incubator for 24 hr to allow adherence.
  Ensure that the plates are level in the incubator to avoid uneven distribution of cells.

#### Filtration of the conditioned medium

14 For conditioned medium removal from the keratinocyte plates from step 10, use a sterile pipet to gently pipet up and down twice in each well, with the plate inclined at a 45° angle, and then transfer the conditioned medium to a sterile container (e.g., a sterile beaker or conical centrifuge tube) labeled for the respective group.
15 After all the conditioned medium has been removed, filter the medium through a 0.22‐µm PES syringe filter attached to a sterile syringe to remove any suspended cells. Use the conditioned medium immediately for melanocyte treatment (see steps 21 and 22).

#### Irradiation of the melanocytes

16 Before manipulating the melanocyte plates from step 13, prepare fresh culture medium for the control groups as in step 1.
  For a total of 50 ml solution, combine 42 ml RPMI‐1640, 7.5 ml FBS, and 0.5 ml antibiotic‐antimycotic.
17 Using separate sterile pipets, remove the culture medium from each well individually and gently wash the wells two times each with 2 ml PBS.
  The washing should be done well by well, adding PBS slowly to the wall of the well to avoid detaching the cells. Carefully remove the PBS after each wash, touching the tip of the pipet to the corner of the well to avoid removing the cells.
18 Add 0.5 ml pure RPMI‐1640 culture medium to each well.
19 Perform irradiation as needed as in steps 7 and 8.
20 After irradiation, carefully remove the pure medium from the wells by tilting the plate at a 45° angle and touching the pipet tip to the corner of the well.

#### Application of the conditioned medium to melanocytes

21 Use the filtered conditioned medium from step 15 to treat the melanocytes according to the respective group: M and iM, culture medium supplemented with FBS and antibiotic‐antimycotic; M‐KCM and iM‐KCM, conditioned medium from non‐irradiated keratinocytes; and M‐iKCM and iM‐iKCM, conditioned medium from irradiated keratinocytes.
22 Perform the selected analysis of the melanocytes at the desired time points.
  In our case, the melanocytes were used for mitochondrial activity analysis at 0, 24, 48, and 72 hr after treatment with conditioned medium and/or irradiation.

## REAGENTS AND SOLUTIONS

### Phosphate‐buffered saline (PBS)

8 g sodium chloride (Synth, cat. no. C1060.01.AH)
0.2 g monobasic potassium phosphate (NEON, cat. no. 01373)
0.2 g potassium chloride (Dinâmica química contemporânea, CAS 7447‐40‐7)
1.15 g dibasic sodium phosphate 7 hydrate PA (Na2HPO4.7H_2_O; Synth, cat. no. F1031.01.AG)
MilliQ H_2_O to 1 L
Dissolve reagents for 5 min with magnetic stirrer
Sterilize by autoclaving
Store ≤1 year at 4°C

## COMMENTARY

### Critical Parameters

The main parameters defined in the protocol were the medium to be used for culture, the vehicle in which the cells were irradiated, and the medium conditioning time. The media tested in our initial work were Dulbecco's Modified Eagle's Medium (DMEM), high‐glucose DMEM, and RPMI‐1640. DMEM was excluded here because, although melanocytes responded well to it, keratinocytes showed reduced growth when cultured in this medium. High‐glucose DMEM and RPMI‐1640 showed similar results in the analyses performed; however, RPMI‐1640 was chosen due to its lighter color, which allowed for higher irradiance during irradiation. The irradiance in the experiments must be measured with consideration of the color of the medium.

Cells were irradiated in culture medium to avoid loss of concentration, as they maintained better adhesion after irradiation compared to when irradiated in PBS. The cells should be irradiated without the presence of FBS to prevent protein degradation and reduction of the irradiance. The UVA radiation dose chosen for our experiments was 4 J/cm²; however, this dose can be adjusted depending on the desired effect. Furthermore, the protocol can also be used to analyze the effects of UVB radiation. To define the most appropriate dose, it is recommended to establish a dose‐response curve for keratinocytes in relation to the selected radiation. For comparative studies between UVA and UVB radiation, it is suggested to use their natural environmental proportions, namely 90% to 95% UVA and 5% to 10% UVB (D'Orazio et al., 2013).

Additionally, the medium conditioning time was maintained at 24 hr, as this resulted in more significant proliferation of treated melanocytes.

### Troubleshooting

Table 1 summarizes a common problem encountered during the protocol, along with a possible cause and a recommended solution.

**Table 1:** Troubleshooting Guide for Preparing Conditioned Medium from Irradiated Keratinocytes

Problem	Possible cause	Solution
Detachment of cells during or after irradiation	Wrong vehicle for irradiation	It is recommended to irradiate in culture medium to protect the cells

### Understanding Results

**Figure 2:**
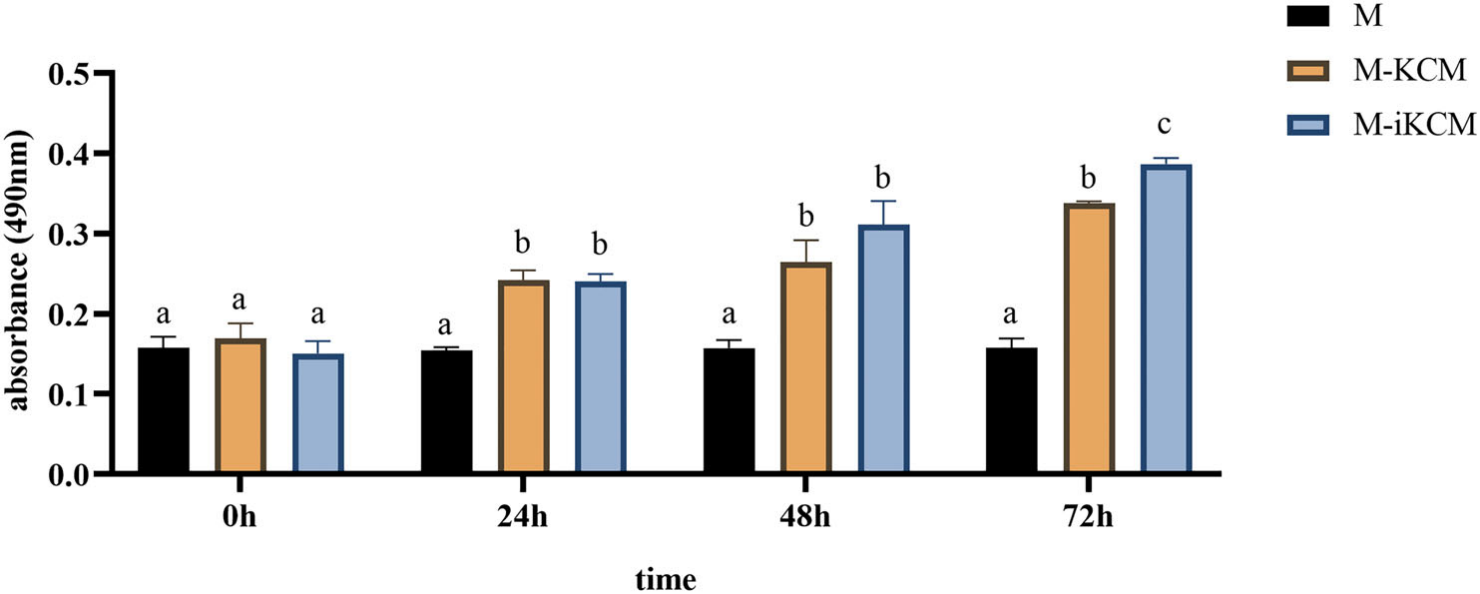
Mitochondrial activity assessed by the MTT assay in murine melanocytes treated with culture medium (M), non‐irradiated keratinocyte‐conditioned medium (M‐KCM), or UVA‐irradiated (4 J/cm²) keratinocyte‐conditioned medium (M‐iKCM). All data are expressed as mean ± standard error. Statistical analysis was performed using one‐way ANOVA followed by Tukey's post hoc test. Different lowercase letters indicate statistically significant differences between treatments within each time point (p<0.05).

Using the above protocol, we demonstrated that the conditioned medium from keratinocytes can induce an increase in melanocyte mitochondrial activity, regardless of whether cell lines from different species were used. These results were observed both in melanocytes treated with conditioned medium from non‐irradiated keratinocytes and in those treated with conditioned medium from UVA‐irradiated keratinocytes, with the latter inducing a greater increase (Fig. 2).

**Figure 3:**
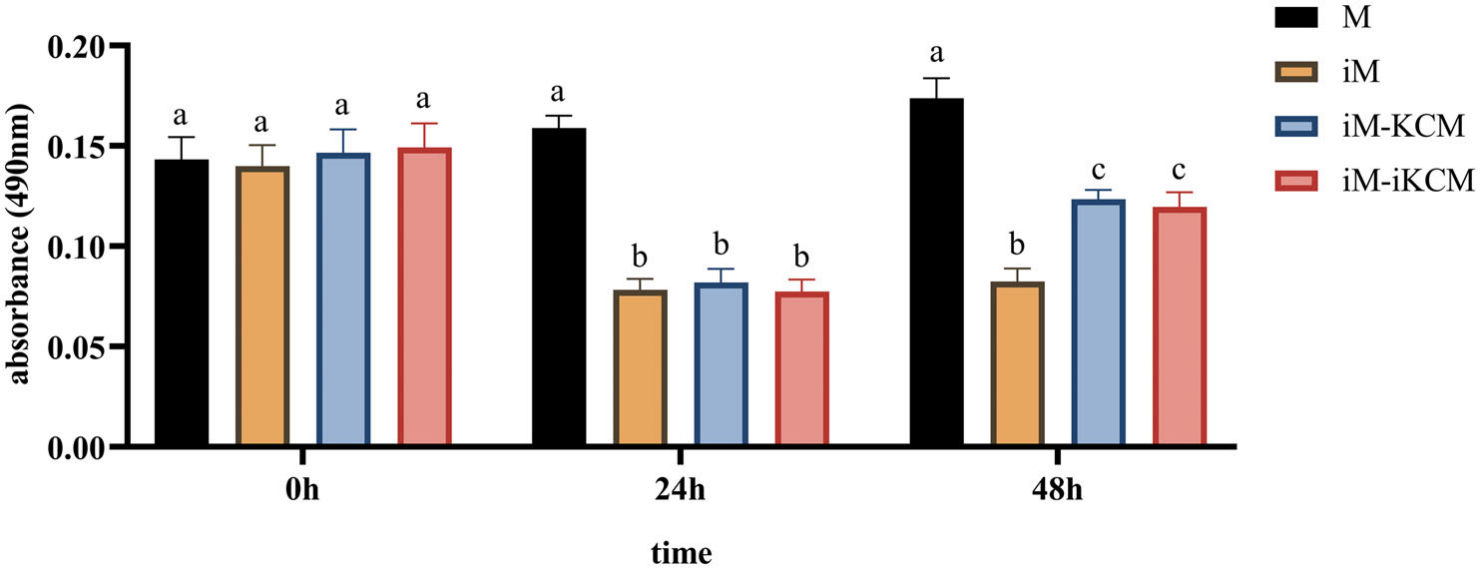
Mitochondrial activity assessed by the MTT assay in murine melanocytes treated with culture medium (M), UVA‐irradiated (4 J/cm²) melanocytes treated with culture medium (iM), UVA‐irradiated (4 J/cm²) melanocytes treated with keratinocyte‐conditioned medium (iM‐KCM), and UVA‐irradiated (4 J/cm²) melanocytes treated with UVA‐irradiated (4 J/cm²) keratinocyte‐conditioned medium (iM‐iKCM). All data are expressed as mean ± standard error. Statistical analysis was performed using one‐way ANOVA followed by Tukey's post hoc test. Different lowercase letters indicate statistically significant differences between treatments within each time point (p<0.05).

Melanocytes directly irradiated with UVA showed a significant decrease in mitochondrial activity after 24 hr of experimentation. However, in the irradiated melanocytes, there was a slight recovery of mitochondrial activity when treated with conditioned medium from either irradiated or non‐irradiated keratinocytes, compared to those treated with regular culture medium (Fig. 3).

### Time Considerations

The protocol described requires 3 days of preparation before the beginning of the analyses. The first day is designated for plating the keratinocytes, requiring ∼2 hr; the second day for irradiating the keratinocytes and plating the melanocytes, requiring ∼4 hr; and the third day for collecting and filtering the conditioned medium and irradiating and treating the melanocytes, requiring ∼5 hr.

### Author Contributions

**Lais Zortéa**: Conceptualization; data curation; formal analysis; investigation; methodology; validation; writing—original draft. **Larissa Gomes Gusmão de Almeida**: Investigation; visualization. **Daza de Moraes Vaz Batista Filgueira**: Conceptualization; data curation; formal analysis; methodology; writing—review and editing. **Ana Paula de Souza Votto**: Conceptualization; funding acquisition; methodology; writing—review and editing; project administration; data curation; supervision; formal analysis; resources.

### Conflict of Interest

The author(s) declare that they have no conflicts of interest relevant to the content of this work. No financial, personal, or professional relationships have influenced the preparation or presentation of this material.

## Data Availability

The data, tools, and material (or their source) that support the protocol are available from the corresponding author upon reasonable request.

## References

[cpz170428-bib-0001] Abdel‐Malek, Z. , & Kadekaro, A. L. (2006). Human pigmentation: Its regulation by ultraviolet light and by endocrine, paracrine, and autocrine factors. In J. J. Nordlund , R. E. Boissy , V. J. Hearing , R. A. King , W. S. Oetting , & J.‐P. Ortonne (Eds.), The pigmentary system: Physiology and pathophysiology, second edition (p. 410–420). John Wiley & Sons. 10.1002/9780470987100.ch20

[cpz170428-bib-0003] Dainese‐Marque, O. , Garcia, V. , Andrieu‐Abadie, N. , & Riond, J. (2024). Contribution of keratinocytes in skin cancer initiation and progression. International Journal of Molecular Sciences, 25(16), 8813. 10.3390/ijms25168813 39201498 PMC11354502

[cpz170428-bib-0005] Diffey, B. L. (2002). Human exposure to solar ultraviolet radiation. Journal of Cosmetic Dermatology, 1(3), 124–130. 10.1046/j.1473-2165.2002.00060.x 17147711

[cpz170428-bib-0006] D'Orazio, J. , Jarrett, S. , Amaro‐Ortiz, A. , & Scott, T. (2013). UV radiation and the skin. International Journal of Molecular Sciences, 14(6), 12222–12248. 10.3390/ijms140612222 23749111 PMC3709783

[cpz170428-bib-0007] Fitzpatrick, T. B. , & Breathnach, A. S. (1963). *The epidermal melanin unit system* [Das epidermale Melanin‐Einheit‐System]. Dermatologische Wochenschrift, 147, 481–489. 14172128

[cpz170428-bib-0008] Gilchrest, B. A. , Park, H. Y. , Eller, M. S. , & Yaar, M. (1996). Mechanisms of ultraviolet light‐induced pigmentation. Photochemistry and Photobiology, 63(1), 1–10. 10.1111/j.1751-1097.1996.tb02988.x 8577860

[cpz170428-bib-0009] Junqueira, L. , & Carneiro, J. (2017). Histologia Básica—Texto e Atlas (13^a^ edição). Guanabara Koogan.

[cpz170428-bib-0010] Li, W. , Pang, Y. , He, Q. , Song, Z. , Xie, X. , Zeng, J. , & Guo, J. (2024). Exosome‐derived microRNAs: Emerging players in vitiligo. Frontiers in Immunology, 15, 1419660. 10.3389/fimmu.2024.1419660 39040109 PMC11260631

[cpz170428-bib-0011] Upadhyay, P. R. , Ho, T. , & Abdel‐Malek, Z. A. (2021). Participation of keratinocyte‐ and fibroblast‐derived factors in melanocyte homeostasis, the response to UV, and pigmentary disorders. Pigment Cell & Melanoma Research, 34(4), 762–776. 10.1111/pcmr.12985 33973367 PMC8906239

